# Epigenomic diversity of colorectal cancer indicated by LINE-1 methylation in a database of 869 tumors

**DOI:** 10.1186/1476-4598-9-125

**Published:** 2010-05-27

**Authors:** Yoshifumi Baba, Curtis Huttenhower, Katsuhiko Nosho, Noriko Tanaka, Kaori Shima, Aditi Hazra, Eva S Schernhammer, David J Hunter, Edward L Giovannucci, Charles S Fuchs, Shuji Ogino

**Affiliations:** 1 Department of Medical Oncology, Dana-Farber Cancer Institute and Harvard Medical School, Boston, MA, USA; 2 Department of Biostatistics, Harvard School of Public Health, Boston, MA, USA; 3 Department of Epidemiology, Harvard School of Public Health, Boston, MA, USA; 4 Channing Laboratory, Department of Medicine, Brigham and Women's Hospital and Harvard Medical School, Boston, MA, USA; 5 Ludwig Boltzmann-Institute for Applied Cancer Research, and Applied Cancer Research - Institution for Translational Research, Vienna, Austria; 6 Department of Nutrition, Harvard School of Public Health, Boston, MA, USA; 7 Department of Pathology, Brigham and Women's Hospital and Harvard Medical School, Boston, MA, USA

## Abstract

**Background:**

Genome-wide DNA hypomethylation plays a role in genomic instability and carcinogenesis. LINE-1 (L1 retrotransposon) constitutes a substantial portion of the human genome, and LINE-1 methylation correlates with global DNA methylation status. LINE-1 hypomethylation in colon cancer has been strongly associated with poor prognosis. However, whether LINE-1 hypomethylators constitute a distinct cancer subtype remains uncertain. Recent evidence for concordant LINE-1 hypomethylation within synchronous colorectal cancer pairs suggests the presence of a non-stochastic mechanism influencing tumor LINE-1 methylation level. Thus, it is of particular interest to examine whether its wide variation can be attributed to clinical, pathologic or molecular features.

**Design:**

Utilizing a database of 869 colorectal cancers in two prospective cohort studies, we constructed multivariate linear and logistic regression models for LINE-1 methylation (quantified by Pyrosequencing). Variables included age, sex, body mass index, family history of colorectal cancer, smoking status, tumor location, stage, grade, mucinous component, signet ring cells, tumor infiltrating lymphocytes, CpG island methylator phenotype (CIMP), microsatellite instability, expression of TP53 (p53), CDKN1A (p21), CTNNB1 (β-catenin), PTGS2 (cyclooxygenase-2), and FASN, and mutations in *KRAS, BRAF*, and *PIK3CA*.

**Results:**

Tumoral LINE-1 methylation ranged from 23.1 to 90.3 of 0-100 scale (mean 61.4; median 62.3; standard deviation 9.6), and distributed approximately normally except for extreme hypomethylators [LINE-1 methylation < 40; N = 22 (2.5%), which were far more than what could be expected by normal distribution]. LINE-1 extreme hypomethylators were significantly associated with younger patients (p = 0.0058). Residual plot by multivariate linear regression showed that LINE-1 extreme hypomethylators clustered as one distinct group, separate from the main tumor group. The multivariate linear regression model could explain 8.4% of the total variability of LINE-1 methylation (R-square = 0.084). Multivariate logistic regression models for binary LINE-1 hypomethylation outcomes (cutoffs of 40, 50 and 60) showed at most fair predictive ability (area under receiver operator characteristics curve < 0.63).

**Conclusions:**

LINE-1 extreme hypomethylators appear to constitute a previously-unrecognized, distinct subtype of colorectal cancers, which needs to be confirmed by additional studies. Our tumor LINE-1 methylation data indicate enormous epigenomic diversity of individual colorectal cancers.

## Introduction

DNA methylation is a major epigenetic mechanism in X-chromosome inactivation, imprinting and repression of transposable elements and endogenous retroviral sequences [1]. Global DNA hypomethylation appears to play an important role in genomic instability [2,3], leading to cancer development [4-8]. Since LINE-1 or L1 retrotransposon constitutes a substantial portion (approximately 17%) of the human genome [9], the methylation status of LINE-1 reflects the global DNA methylation level. Prior studies have shown that tumor LINE-1 methylation correlates with cellular 5-methylcytocine level in cancer tissues [10-12].

In addition to the role as a surrogate marker for global DNA methylation, LINE-1 methylation status by itself likely has biological effects, since retrotransposons such as LINE-1 elements can provide alternative promoters [13], and contribute to non-coding RNA expression that regulates functions of a number of genes [14,15]. Moreover, retrotransposons activated by DNA hypomethylation may transpose themselves throughout the genome, leading to gene disruptions [16] and chromosomal instability [4,17]. LINE-1 methylation has been shown to be highly variable among colon cancers [18,19], and LINE-1 hypomethylation is strongly associated with poor prognosis in colon cancer [20]. However, whether LINE-1 hypomethylators constitute a distinct tumor subtype remains uncertain. Our recent study has shown a significant correlation of LINE-1 methylation levels within synchronous colorectal cancer pairs (i.e., two or more primary tumors in one patient), which suggests the presence of genetic and/or environmental factors influencing LINE-1 methylation levels that are unlikely the result of a purely stochastic phenomenon [21]. Therefore, there are two distinct phenomena - a wide variation of LINE-1 methylation in colorectal cancers and a significant concordance of LINE-1 methylation within synchronous colorectal cancer pairs - raising the intriguing question of whether this wide variation of tumoral LINE-1 methylation can be explained by various clinical, pathologic or molecular variables.

To address this question, we conducted this study with a large database (N = 869) of colorectal cancers identified in two prospective cohort studies and multivariate linear and logistic regression models for LINE-1 methylation level using clinical, pathologic and molecular variables. We have found that the variability in LINE-1 methylation levels remains even after accounting for clinical, pathologic and molecular variables, which indicates epigenomic diversity of colorectal cancers. We have also found that LINE-1 extreme hypomethylators might constitute a previously-unrecognized, distinct cancer subtype, which may have substantial clinical implications with its young age of onset and aggressive behavior [20].

## Materials and methods

### Study group

We utilized the databases of two large prospective cohort studies, the Nurses' Health Study (N = 121,700 women followed since 1976) [22], and the Health Professional Follow-up Study (N = 51,529 men followed since 1986) [22]. Data on height, weight, smoking status, and family history of colorectal cancer in any first-degree relative were obtained by biennial questionnaire. A subset of the cohort participants developed colorectal cancers during prospective follow-up. Previous studies on these cohort studies have described baseline characteristics of participants and incident colorectal cancer cases, and confirmed that our colorectal cancers were well representative as a population-based sample [22]. Data on tumor location and stage were obtained through medical record review. We collected paraffin-embedded tissue blocks from hospitals where patients had undergone resections of colorectal cancers. Based on availability of adequate tissue specimens and results, a total of 869 colorectal cancers were included (Table 1). Among our cohort studies, there was no significant difference in demographic features between cases with tissue available and those without available tissue [22]. This current analysis represents a new study using linear and logistic regression models for LINE-1 methylation on the existing colorectal cancer database (which has been previously characterized for molecular features and clinical outcome [19-24]), leading to the discovery of a possibly novel subtype of colorectal cancers. We comprehensively included many of the previously characterized tumor markers [19-24]), which have been linked to colorectal carcinogenesis. In our previous studies which focused on different hypotheses, we examined the relationship between LINE-1 methylation and CpG island methylator phenotype (CIMP) to test the hypothesis whether there is any relationship between global DNA methylation level and CIMP [19]; the relation between LINE-1 methylation and patient survival [20]; the relationship between LINE-1 methylation levels (as one of many molecular markers) within synchronous colorectal cancer pairs [21]; and the relationship between LINE-1 hypomethylation and 18 q loss of heterozygosity (LOH) as a part of analysis on prognostic significance of 18 q LOH [23]. Informed consent was obtained from all study subjects. Tissue collection and analyses were approved by the Harvard School of Public Health and Brigham and Women's Hospital Institutional Review Boards.

**Table 1:** LINE-1 methylation level in colorectal cancer.

Clinical, pathologic or molecular feature	Total N	Mean	Standard deviation	P value^
All cases	869	61.4	9.6	
Sex				0.19
Men	394 (45%)	61.0	9.0	
Women	475 (55%)	61.8	10.1	
Age (years)				0.35
< 60	196 (23%)	60.5	10.5	
60-69	363 (42%)	61.7	8.7	
≥70	310 (36%)	61.5	9.9	
Body mass index (BMI, kg/m^2^)				0.96
< 30	667 (83%)	61.3	9.5	
≥30	141 (17%)	61.4	9.1	
Family history of colorectal cancer				0.005
(-)	657 (76%)	61.9	9.6	
(+)	212 (24%)	59.7	9.1	
Smoking status				0.99
Never	350 (41%)	61.4	10.1	
Past or current	508 (59%)	61.3	9.2	
Tumor location				0.024
Proximal colon (cecum to transverse)	370 (44%)	61.9	9.2	
Distal colon (splenic flexure to sigmoid)	274 (33%)	60.1	9.7	
Rectum	192 (23%)	62.3	9.4	
Disease stage				0.015
I	196 (25%)	61.5	9.2	
II	253 (33%)	62.5	9.2	
III	226 (29%)	60.1	9.5	
IV	103 (13%)	60.0	11.0	
Tumor grade				0.43
Low	777 (91%)	61.3	9.4	
High	79 (9.2%)	62.3	11.2	
Mucinous component				0.002
0%	554 (65%)	60.7	9.3	
1-49%	188 (22%)	61.3	10.0	
≥50%	115 (13%)	64.1	9.3	
Signet ring cell component				0.009
0%	796 (93%)	61.0	9.4	
1-49%	48 (5.6%)	63.5	10.8	
≥50%	14 (1.6%)	67.6	8.5	
Crohn's-like reaction				0.002
Absent/mild	684 (92%)	61.1	9.5	
Moderate/severe	62 (8.3%)	65.2	9.6	
Peritumoral lymphocytic reaction				0.018
Absent/mild	744 (89%)	60.9	9.4	
Moderate/severe	93 (11%)	63.6	10.3	
Tumor infiltrating lymphocytes (TIL)				0.043
Absent/mild	740 (89%)	60.9	9.5	
Moderate/severe	96 (11%)	63.2	10.2	
MSI status				< 0.0001
MSI-low/MSS	728 (85%)	60.7	9.5	
MSI-high	124 (15%)	64.7	8.9	
CIMP status				< 0.0001
CIMP-0	408 (47%)	60.2	9.8	
CIMP-low	333 (38%)	61.3	9.3	
CIMP-high	128 (15%)	65.1	8.3	
CIN status (in MSI-low/MSS cases)				0.0002
(-)	142 (25%)	63.7	9.6	
(+)	436 (75%)	60.3	9.0	
*KRAS *mutation				0.90
(-)	538 (63%)	61.3	9.6	
(+)	319 (37%)	61.2	9.4	
*BRAF *mutation				0.001
(-)	728 (87%)	60.8	9.3	
(+)	108 (13%)	64.2	10.2	
*PIK3CA *mutation				0.24
(-)	646 (85%)	61.1	9.7	
(+)	118 (15%)	62.2	9.3	
TP53 expression				0.096
(-)	488 (57%)	61.8	9.8	
(+)	371 (43%)	60.7	9.3	
CDKN1A (p21)				0.0002
Lost	682 (81%)	60.6	9.6	
Expressed	158 (19%)	63.7	9.0	
CTNNB1 (β-catenin) score*				0.47
0-2 (inactive)	482 (64%)	61.4	9.8	
3-5 (active)	273 (36%)	60.9	9.3	
PTGS2 (COX-2) expression				0.98
(-)	142 (16%)	61.3	11.2	
(+)	719 (84%)	61.3	9.2	
FASN expression				0.16
(-)	742 (88%)	61.1	9.7	
(+)	99 (12%)	62.4	8.7	

^ P value was calculated by t-test assuming unequal variances (for binary variables) or ANOVA (analysis of variance; for 3-category variables). * CTNNB1 score was calculated as previously described [24].

There were cases with missing information in covariates. A p value for significance is adjusted to p = 0.0021 by Bonferroni correction for multiple hypothesis testing.

CIMP, CpG island methylator phenotype; MSI, microsatellite instability; MSS, microsatellite stable.

### Histopathologic evaluations

Hematoxylin and eosin (H&E) stained tissue sections were examined by a pathologist (S.O.) unaware of other data. The tumor grade was categorized as low (≥ 50% gland formation) vs. high (< 50% gland formation) [25]. The presence/absence and extent of extracellular mucin and signet ring cells were recorded as percentage of mucinous and signet ring cell components, respectively [25]. Each of Crohn's-like lymphocytic reaction, peritumoral lymphocytic reaction and tumor infiltrating lymphocytes (TIL) was recorded as either absent, mild, moderate or severe [25].

### DNA extraction and sodium bisulfite treatment

The methods for sodium bisulfite treatment of DNA have previously been described [26]. H&E-stained slides of the tumors were reviewed, and areas of tumor were marked, to exclude pure normal tissue and enrich tumor DNA. Tissue sections (depending on tissue and tumor size, in average, large tumor tissue 10 μm × 1 section) from each case were scraped off slides, suspended in 20 μl of the tissue lysate (100 mmol/L Tris, pH 8, 10 mmol/L ethylenediaminetetraacetic acid, pH 8, 1 mg/ml proteinase K, and 0.05 mg/ml tRNA), and incubated at 50°C overnight. The tissue lysate was then stored at -20°C until sodium bisulfite treatment was performed. Sodium metabisulfite (1.9 g) was dissolved in mixture of 3.2 ml of 0.44 mol/L NaOH at 50°C. Then, 0.5 ml of 1 mol/L hydroquinone was added to the dissolved sodium bisulfite mixture. An 18-μl aliquot of the tissue lysate was denatured at 100°C for 10 minutes and chilled on ice. Then, 2 μl of 3 mol/L NaOH was added and incubated at 42°C for 20 minutes. The bisulfite solution (120 μl) was added (total volume of 140 μl) and incubated at 50°C for 15 hours in the dark. The bisulfite-converted DNA was recovered using a Qiagen QIAamp viral RNA mini kit (Qiagen, Valencia, CA) according to the manufacturer's instructions with some modifications. Buffer AVL/carrier RNA (560 μl) was added to the 140 μl of bisulfite-converted DNA sample and incubated at room temperature for 10 minutes. Ethanol (560 μl) was then added, and after extensive mixing, the mixture was loaded onto the provided spin columns in consecutive 630-μl aliquots. After each loading, the columns were centrifuged at full speed (21,000 × *g*) for 1 minute. Both the filtrate and spin column were saved, and both filtrates were passed through the column a second time in the same manner to increase the yield of recovery. The spin column was then washed with 500 μl of buffer AW1, followed by centrifugation at 21,000 × *g *for 1 minute. Buffer AW2 (500 μl) was then added to the column, and the column was centrifuged at 21,000 × *g *for 4 minutes to wash the column and eliminate possible buffer AW2 carry over. DNA in the spin column was eluted by the addition of 40 μl of buffer AVE, followed by a 1-minute incubation at room temperature and centrifugation at 7600 × *g *for 1 minute. This elution step was repeated with a second 40-μl volume of buffer AVE. Fifty μl of 0.2 mol/L NaOH was added to the 80-μl pooled eluate for 15 minutes at room temperature to desulphonate the sample, and then 10 μl of 1 mol/L HCl was added to for neutralization. Buffer AVL/carrier RNA (560 μl) was then added to the 140-μl sample mixture, and the recovery procedure was repeated with a new spin column. The eluted DNA (80-μl volume) was then used for Pyrosequencing analysis and MethyLight analysis.

### Real-time PCR (MethyLight) for quantitative DNA methylation analysis

Subsequent real-time PCR (MethyLight) [27] was validated and performed as previously described [26]. We quantified DNA methylation in 8 CIMP-specific promoters [*CACNA1G, CDKN2A *(p16), *CRABP1, IGF2, MLH1, NEUROG1, RUNX3 *and *SOCS1*] [28-30]. CIMP-high was defined as the presence of ≥ 6 of 8 methylated promoters, CIMP-low as the presence of 1/8-5/8 methylated promoters, and CIMP-0 as the absence (0/8) of methylated promoters, according to the previously established criteria [30,31].

### Pyrosequencing to Measure LINE-1 Methylation

**Figure 1:**
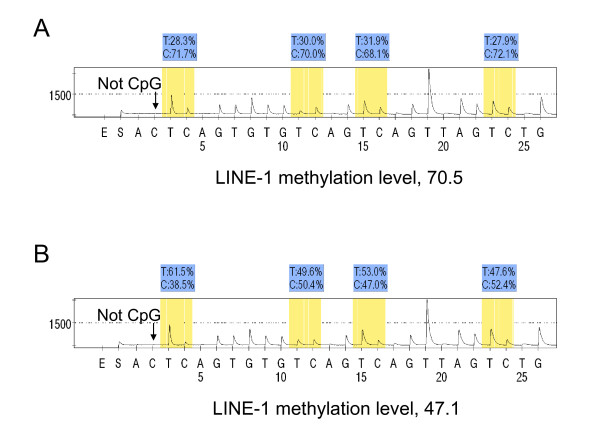
**Pyrosequencing to measure LINE-1 methylation**. A. LINE-1 hypermethylated tumor. B. LINE-1 hypomethylated tumor. The % numbers (in blue shade) are proportions of C and T at each CpG site after bisulfite conversion, and the methylation level of each CpG site is estimated by the proportion of C (%). An overall LINE-1 methylation level is calculated as the average of the proportions of C (%) at the 4 CpG sites. The first, third and fourth CpG sites follow mononucleotide T repeats, resulting in higher T peaks (in yellow shade) than the second CpG site, and the proportion of C (%) has been adjusted accordingly. The arrows indicate no residual C at the non-CpG site, ensuring complete bisulfite conversion.

In order to accurately quantify relatively high LINE-1 methylation levels, we utilized Pyrosequencing technology (Figure 1) [10,19,32]. PCR and subsequent Pyrosequencing for LINE-1 were performed using the PyroMark kit (Qiagen). This assay amplifies a region of LINE-1 element (position 305 to 331 in accession No. X58075), which includes 4 CpG cites. The PCR condition was 45 cycles of 95°C for 20 sec, 50°C for 20 sec and 72°C for 20 sec, followed by 72°C for 5 min. The biotinylated PCR product was purified and made single-stranded to act as a template in a pyrosequencing reaction, using the Pyrosequencing Vacuum Prep Tool (Qiagen). Pyrosequencing reactions were performed in the PSQ HS 96 System (Qiagen). The nucleotide dispensation order was: ACT CAG TGT GTC AGT CAG TTA GTC TG. The non-CpG cytosine in LINE-1 repetitive sequences has been documented to be rarely methylated [33]. Thus, complete conversion of cytosine at a non-CpG site ensured successful bisulfite conversion. The amount of C relative to the sum of the amounts of C and T at each CpG site was calculated as percentage (i.e., 0 to 100). The average of the relative amounts of C in the 4 CpG sites was used as overall LINE-1 methylation level in a given tumor. LINE-1 methylation level measured by Pyrosequencing is a good indicator of cellular 5-methylcytosine level (i.e., global DNA methylation level) [10,18]. We compared results from cancer cells collected by laser capture microdissection (LCM) with results from cancer tissues dissected manually using HE sections with marking for tumor areas [32]. We showed that DNA hypomethylation could be measured in manually dissected cancer tissue without LCM, and that precision of measurement in manually dissected cancer tissue was superior to cancer cells collected by LCM [32]. In addition, to extensively validate LINE-1 methylation Pyrosequencing assay, we performed bisulfite conversion on seven different DNA specimen aliquots and repeated PCR-Pyrosequencing seven times using 10 macro-dissected colorectal cancers. Bisulfite-to-bisulfite (between-bisulfite treatment) standard deviation ranged from 0.4-2.4 (median, 1.2), and run-to-run (between-PCR Pyrosequencing run) standard deviation ranged from 1.3-4.4 (median, 3.0) [32]. Moreover, intraclass correlation coefficient (ICC) of LINE-1 methylation in cancer tissues was 0.77, which suggests good reliability in the measurement.

### Sequencing of *KRAS*, *BRAF *and *PIK3CA*, and analyses for microsatellite instability (MSI) and chromosomal instability (CIN)

PCR and Pyrosequencing targeted for *KRAS *(codons 12 and 13) [34], *BRAF *(codon 600) [35] and *PIK3CA *(exons 9 and 20) [36,37] were performed as previously described. MSI analysis was performed, using D2S123, D5S346, D17S250, BAT25, BAT26, BAT40, D18S55, D18S56, D18S67 and D18S487 [38]. MSI-high was defined as the presence of instability in ≥30% of the markers, and MSI-low/microsatellite stability (MSS) as the presence of 0-29% unstable markers. Loss of heterozygosity (LOH) analysis was performed using microsatellite markers (D2S123, D5S346, D17S250, D18S55, D18S56, D18S67 and D18S487). LOH at each locus was defined as ≥40% reduction of one of two allele peaks in tumor DNA relative to normal DNA. CIN positivity was defined as the presence of LOH in any of the chromosomal segments among 2 p, 5 q, 17 q and 18 q, and CIN negativity was defined as the absence of LOH in any of the chromosomal segments with the presence of at least two informative segments [39,40].

### Immunohistochemistry for TP53, CDKN1A, CTNNB1, PTGS2 and FASN

Tissue microarrays (TMA) were constructed as previously described [41]. Methods of immunohistochemistry were previously described for TP53 (p53) [42], CDKN1A (p21) [43,44]; CTNNB1 (β-catenin) [24]; PTGS2 (cyclooxygenase-2; COX-2) [22,38] and FASN [38,45] (Additional file 1 for the method summary). The CTNNB1 score (i.e., a summation of membrane loss, and cytoplasmic and nuclear localization) used in this current study has been used as a surrogate of CTNNB1 activation caused not only by *APC *loss but also by other mechanisms, including *APC *mutation, *APC *methylation, and *CTNNB1 *mutation [46]. Appropriate positive and negative controls were included in each run of immunohistochemistry. Each immunohistochemical maker was interpreted by one of the investigators (TP53, CDKN1A, PTGS2 and FASN by S.O.; CTNNB1 by K.N.) unaware of other data. A random selection of 108-179 cases was examined for each marker by a second observer (TP53 and FASN by K.N.; CDKN1A by K.S.; CTNNB1 by S.O.; PTGS2 by R. Dehari, Kanagawa Cancer Center) unaware of other data. The κ coefficients between the two observers were 0.65 for CTNNB1 (N = 142), 0.62 for CDKN1A (N = 179), 0.75 for TP53 (N = 118), 0.57 for FASN (N = 146) and 0.62 for PTGS2 (N = 108) (all p < 0.0001), indicating good to substantial agreement.

### Statistical analysis

We used the SAS program (Version 9.1, SAS Institute, Cary, NC) for all statistical analyses. All p values were two-sided. Because of multiple hypothesis testing, a p value for significance was adjusted by Bonferroni correction to p = 0.0021 (= 0.05/24). To compare mean LINE-1 methylation levels, we performed the t-test assuming unequal variances or ANOVA for variables with more than 2 categories. Pearson correlation test was used to assess correlations of LINE-1 methylation with raw continuous values of age, body mass index (BMI), percentage of mucinous component and percentage of signet ring cell component. Fisher's exact test was used to assess associations between categorical variables.

We constructed a multivariate linear regression model to assess whether clinical, pathologic and molecular variables could predict LINE-1 methylation level in 869 colorectal cancers. Variables initially included sex, age (continuous), BMI (continuous), smoking status (continuous pack-years), family history of colorectal cancer in any first degree relative (present vs. absent), tumor location (proximal colon vs. distal colon vs. rectum), disease stage (I vs. II vs. III vs. IV vs. unknown), tumor grade (low vs. high), mucinous component (%, continuous), signet ring cell component (%, continuous), Crohn's-like reaction (ordinal scale 1-4), peritumoral lymphocytic reaction (ordinal scale 1-4), tumor infiltrating lymphocytes (ordinal scale 1-4), CTNNB1 score (ordinal scale 1-5), CIMP status (high vs. low/0), MSI status (high vs. low/MSS), *BRAF *(mutant vs. wild-type), *KRAS *(mutant vs. wild-type), *PIK3CA *(mutant vs. wild-type), TP53 (positive vs. negative), CDKN1A (expressed vs. lost), PTGS2 (positive vs. negative), and FASN (positive vs. negative). Cases with missing data in a categorical or ordinal variable were included in a majority (or the most common) category; such variables included BMI (7.0%), tumor location (3.8%), stage (10%), grade (1.5%), mucinous component (1.4%), signet ring cell component (1.3%), peritumoral lymphocytic reaction (3.7%), TIL (3.8%), MSI (2.2%), *KRAS *(1.4%), *BRAF *(3.8%), *PIK3CA *(12%), TP53 (1.2%), CDKN1A (3.4%), CTNNB1 (13%), PTGS2 (0.9%) and FASN (3.2%). We performed multivariate linear regression analysis with a backward stepwise elimination procedure to limit the variables with p ≤ 0.20. After the final multivariate linear regression model was constructed, a distribution of residuals (observed LINE-1 methylation level minus predicted LINE-1 methylation level by the regression model) was visually inspected and confirmed that the assumptions of residuals' normality and equal variance across LINE-1 methylation level were generally satisfied. We assessed whether there were any individually influential outliers by residual plots and Cook's D statistics (a summary measure of influence) and found that there were no significant outliers (Cook's D value < 0.035 for all cases). This indicates that our overall findings were not influenced by any particular outlier subjects.

We assessed potential non-linearity of continuous and ordinal variables (age, BMI, smoking status, disease stage, mucinous component, signet ring cell component, Crohn's-like reaction, peritumoral lymphocytic reaction, tumor infiltrating lymphocytes and CTNNB1 score) by constructing a squared term for each of these variables (excluding data-missing cases) and included these with the original variables in an additional multivariate model. As a result, no squared term showed significant relationship with LINE-1 methylation (all p > 0.20).

In addition, we constructed a multivariate logistic regression model for the binary LINE-1 outcome (cutoff at 40, 50 or 60) using the same set of covariates as in the final linear regression model. The logistic regression model formula yielded a score for each case based on β coefficients and a combination of covariate status. We drew ROC (receiver operator characteristics) curves for the diagnosis of LINE-1 hypomethylation as the binary outcome, using different thresholds of the regression score for test positivity. ROC curves for each cutoff was averaged over 10 folds each using 25% randomly heldout test data for cross validation. Area under the curve (AUC) value of an averaged ROC curve reflected an ability of a regression model to diagnose binary LINE-1 hypomethylation.

## Results

### LINE-1 methylation levels in 869 colorectal cancers

Utilizing 869 colorectal cancers identified in the two independent prospective cohort studies, we quantified LINE-1 methylation by bisulfite-PCR and Pyrosequencing technology (Figure 1). LINE-1 methylation is a good indicator of cellular methylcytosine level (i.e., global DNA methylation level) [10-12], and the Pyrosequencing assay described here can provide precise data for LINE-1 methylation levels [10,12,18,32]. Using the 4 CpG sites in LINE-1, we calculated the average of the percentage numbers (at the 4 CpG sites) of C (methylated) allele among C (methylated) and T (unmethylated) alleles and used this as the LINE-1 methylation level in a given tumor (described as 0 to 100). We previously examined LINE-1 methylation levels in normal colonic mucosa adjacent to colorectal cancers as well as peripheral blood cells from normal individuals, and found that LINE-1 methylation levels in those specimens were high (> 65) [21,32]. In addition, we previously showed that DNA hypomethylation could be precisely measured in manually dissected cancer tissue, and that precision of LINE-1 methylation measurement in manually dissected cancer tissue was superior to LINE-1 methylation measurement in cancer cells collected by laser capture microdissection (LCM) [32]. Because a manual dissection procedure could be easily implemented in this large-scale study and a large amount of DNA could be obtained for better precision, we used manual dissection to obtain DNA from the 869 tumors.

**Figure 2:**
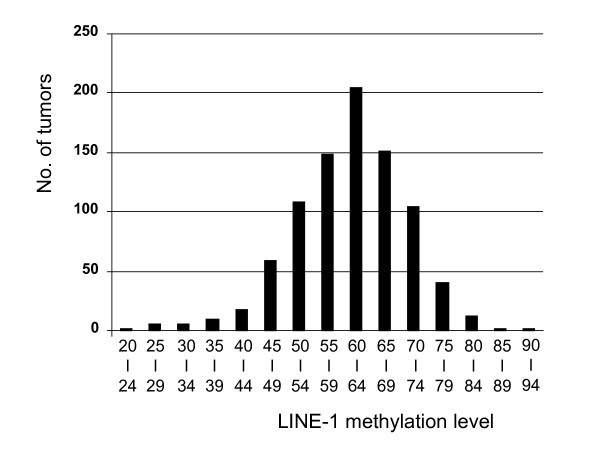
**Distribution of LINE-1 methylation levels in 869 colorectal cancers**. LINE-1 methylation levels distributed approximately normally, except for the presence of extreme hypomethylators (LINE-1 methylation value < 40), which appear to be separate from the remaining normal distribution.

LINE-1 methylation levels in the 869 tumors (Figure 2) widely distributed (ranging from 23.1 to 90.3 of 0-100 scale; median 62.3; mean 61.4; interquartile range 12.5) and fell into two apparent classes: 22 extreme hypomethylators below a methylation level of 40, and 847 remaining tumors with normally distributed LINE-1 methylation levels (Shapiro-Wilk p > 0.20 for a deviation of normality). We have shown that between-assay variation of LINE-1 methylation levels in repeated measurements is small and coefficient of variation (CV) is 4-5% [32]; this argues against attributing the large variation in LINE-1 methylation observed between tumors to a laboratory measurement error. In combination with the highly significant association between LINE-1 hypomethylation and patient mortality [20], this indicates that variation in LINE-1 methylation is likely intrinsic to the biology of individual tumors.

### LINE-1 methylation and clinical, pathologic and molecular variables

We examined LINE-1 methylation level in colorectal cancer according to various clinical, pathologic or molecular feature (Table 1). In univariate analysis (with Bonferroni-corrected significance level at p = 0.05/24 = 0.0021), LINE-1 methylation was significantly related with mucinous component (p = 0.002), Crohn's-like reaction (p = 0.002), microsatellite instability (MSI, p < 0.0001), CpG island methylator phenotype (CIMP, p < 0.0001), chromosomal instability (CIN) in MSI-low/MSS cases (p = 0.0002), *BRAF *mutation (p = 0.001) and CDKN1A (p21) expression (p = 0.0002).

We also examined correlations of LINE-1 methylation with raw values for age, body mass index (BMI), percentage of mucinous component and percentage of signet ring cell component. In all cases, the Pearson correlation coefficient was between 0 and 0.164 (i.e., all R^2 ^< 0.027), indicating that none of these variables alone could explain substantial variability of LINE-1 methylation.

### Multivariate linear regression analysis for tumoral LINE-1 methylation level

We constructed a multivariate linear regression model for LINE-1 methylation level as an outcome variable, using clinical, pathologic and other molecular variables. A model that could predict LINE-1 methylation level might be useful, considering the importance of LINE-1 hypomethylation as a highly significant prognostic biomarker in colon cancer [20]. Variables in the multivariate linear regression model initially included sex, age, BMI, family history of colorectal cancer, smoking status, disease stage tumor location, grade, mucinous component, signet ring cells, Crohn's-like reaction, peritumoral lymphocytic reaction, tumor infiltrating lymphocytes (TIL), CIMP, MSI, *BRAF *mutation, *KRAS *mutation, *PIK3CA *mutation, TP53 expression, CDKN1A expression, CTNNB1 score, PTGS2 expression, and FASN expression. We excluded CIN status from our list of covariates because global DNA hypomethylation might be a cause of CIN. Thus, in a linear regression model to predict LINE-1 methylation level (as an outcome variable), a putative consequential variable (such as CIN) should not be put as a predictor variable. After a backward stepwise elimination to avoid overfitting, the variables listed in Table 2 remained in the final model. Importantly, the R-square of the final multivariate model was only 0.084, indicating that 92% of the LINE-1 methylation variability still remained.

**Table 2:** Multivariate linear regression analysis to predict LINE-1 methylation level in 869 colorectal cancers

Variables in the final model	Adjusted β coefficient (change in LINE-1 methylation level by a given variable)	95% confidence limits	P value (partial F-test)
CIMP status			< 0.0001
CIMP-high (vs. CIMP-0)	4.79	2.69, 6.89	< 0.0001 (T test)
CIMP-low (vs. CIMP-0)	1.17	-0.18, 2.52	0.090 (T test)
Signet ring cell component (for 10% increase)	1.15	0.53, 1.77	0.0003
Rectal location (vs. colon)	2.22	0.68, 3.75	0.0046
Family history of colorectal cancer (present vs. absent)	-1.92	-3.36, -0.48	0.0089
Disease stage (for one unit increase in ordinal scale, I-IV)	-0.83	-1.50, -0.16	0.016
CDKN1A (p21) loss (vs. expression)	-2.06	-3.75, -0.37	0.017
Crohn's-like reaction [for one unit increase in ordinal scale 1 (absent)-4 (strong)]	1.26	-0.20, 2.31	0.020
High tumor grade (vs. low grade)	-2.47	-5.00, 0.07	0.056
Male (vs. female)	1.03	-0.24, 2.29	0.11
Body mass index (BMI, for an increase of 5 kg/m^2^)	0.50	-0.18, 1.18	0.15

The multivariate linear regression model initially included the variables listed in the table, age, smoking, mucinous component, peritumoral lymphocytic reaction, tumor infiltrating lymphocytes, CTNNB1 score, MSI status, mutations in *BRAF*, *KRAS *and *PIK3CA*, and expression status of TP53, CDKN1A (p21), PTGS2 (cyclooxygenase-2), and FASN. A backward elimination with a threshold of p = 0.20 was performed to select variables in the final model. The adjusted β coefficient represents a change (increase or decrease) in LINE-1 methylation level by a given variable, assuming that all other variables remain constant. R-square of the multivariate model was only 0.084, indicating that 92% of variability in LINE-1 methylation levels remained unexplained by this model. A p value for significance is adjusted to p = 0.0021 by Bonferroni correction for multiple hypothesis testing.

CIMP, CpG island methylator phenotype; MSI, microsatellite instability.

**Figure 3:**
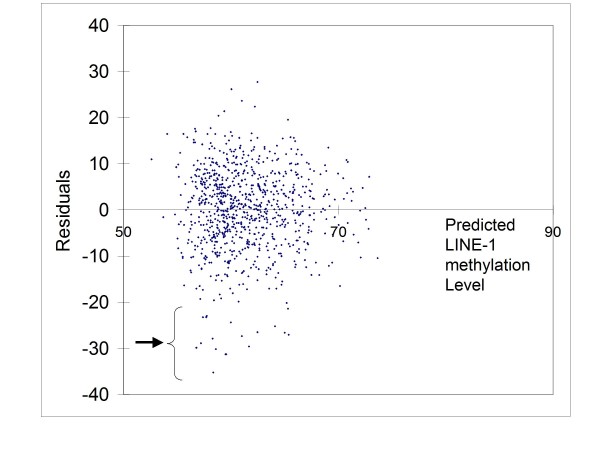
**Residuals plot of multivariate linear regression model to predict LINE-1 methylation in 869 colorectal cancers**. Each dot represents each case. The x axis represents LINE-1 methylation level predicted by the regression model, and the y axis represents the residuals (observed LINE-1 methylation level minus predicted LINE-1 methylation level by the regression model). A distribution of the residuals is approximately normal across predicted LINE-1 methylation levels, except for the presence of outliers (arrow). The arrow suggests the presence of a distinct group of tumors, separate from the normal and homoscedastic distribution around the 0 residual line.

To validate the multivariate linear regression model, we examined the residuals (i.e., observed LINE-1 methylation level minus predicted LINE-1 methylation level by the regression model) (Figure 3). Across all predicted LINE-1 methylation levels, the distribution of residuals was approximately normal and homoscedastic. These results suggested that the multivariate linear regression model was in general appropriately constructed. Of note, there was a cluster of tumors which were separate from the normal and homoscedastic distribution around the 0 residual line. These tumors greatly overlapped with LINE-1 extreme hypomethylators (LINE-1 methylation level < 40).

**Figure 4:**
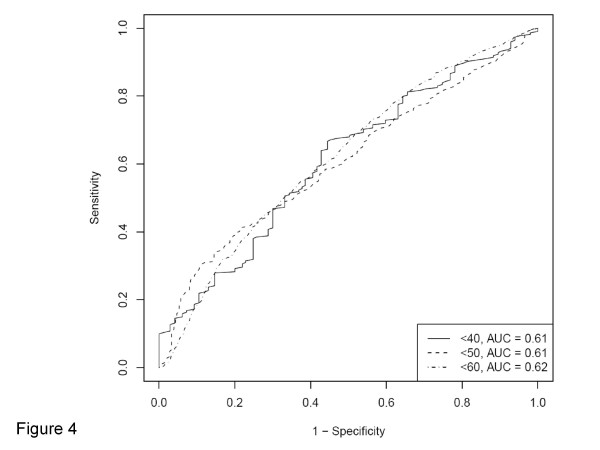
**ROC (receiver operator characteristics) curves for the diagnosis of LINE-1 hypomethylation as a binary outcome using different cutoffs (40, 50 and 60 of 0-100 scale)**. Multivariate logistic regression models for the binary LINE-1 methylation outcomes were constructed using the same set of covariates as in the linear regression model. ROC curves for each cutoff was averaged over 10 folds each using 25% randomly heldout test data for cross validation. Area under the curve (AUC) represents performance characteristics of the multivariate model as a clinical test for the specific binary outcome.

All variables included in the final multivariate linear regression model are listed in Table 2. The adjusted β coefficient represented an increase in LINE-1 methylation level by a given variable, assuming that all other variables remained constant. The most significant predictor was CIMP-high [vs. CIMP-0; adjusted β coefficient 4.79; 95% confidence interval (CI), 2.69 to 6.89; p < 0.0001], followed by signet ring cells (for 10% increase; adjusted β coefficient 1.15; 95% CI, 0.53 to 1.77; p = 0.0003). Based on the conservative significance level at p = 0.0021 by Bonferroni correction for multiple hypothesis testing, no other variables showed robustly significant association, although major indicators including family history, tumor location, and stage showed nominal significance (insufficient to contribute significantly to model accuracy; see Figure 4). All other variables were not significantly associated with LINE-1 methylation.

To eliminate potential confounding effect of HNPCC (hereditary nonpolyposis colorectal cancer), we identified 19 possible or suspected HNPCC cases [i.e., MSI-high CIMP-low/0 tumors (none of which turned out to be *BRAF*-mutated) with any of the followings: (1) positive family history of colorectal cancer in at least one first-degree relative; (2) loss of MLH1 without evidence of *MLH1 *methylation; (3) loss of PMS2 without evidence of MLH1 loss; (4) loss of MSH2 and/or MSH6]. After we excluded these 19 cases, multivariate linear regression analysis showed very similar results (data not shown).

### ROC curves for binary LINE-1 methylation outcome in logistic regression analysis

We constructed multivariate logistic regression models for binary LINE-1 outcomes using different cutoffs (40, 50 and 60) with the same set of covariates as in the final linear regression model. The logistic regression model formula yielded a score for each case based on β coefficients and a combination of covariate status. We drew ROC (receiver operator characteristics) curves for the diagnosis of LINE-1 hypomethylation as a binary outcome, using different thresholds of the regression score for test positivity (Figure 4). Area under the curve (AUC) remained relatively low (< 0.63) for LINE-1 hypomethylation at a cutoff of < 40, < 50 or < 60 LINE-1 methylation. These results also support our finding that LINE-1 methylation status was not well predicted by these variables.

### LINE-1 extreme hypomethylated tumors

We examined clinical, pathologic and molecular features of tumors with LINE-1 methylation value below 40, because both the overall distribution of LINE-1 methylation levels (Figure 2) and the residuals plot of the linear regression model (Figure 3) indicated the presence of a distinct group of LINE-1 extreme hypomethylators. Notably, there was a relationship between LINE-1 extreme hypomethylation and younger age at diagnosis; 45% (10/22) of cases with LINE-1 methylation < 40 were less than 60 years old, while only 22% (186/847) of the remaining patients were less than 60 years old (p = 0.0058) (Table 3). Overall distribution of age in these LINE-1 < 40 hypomethylators was somewhat bimodal with fewer cases (14% = 3/22) being 60-69 years old, compared to the remaining patients (with LINE-1 methylation ≥ 40) where 43% (360/847) of patients were 60-69 years old. While the total number of LINE-1 < 40 hypomethylators (N = 22) was not enough to achieve statistical significance after Bonferroni correction, this difference in age distribution further supports the presence of a distinct group of LINE-1 extreme hypomethylators. These LINE-1 extreme hypomethylators might arise through a different carcinogenic pathway, resulting in a younger age of onset and more aggressive tumor behavior [20].

**Table 3:** LINE-1 extreme hypomethylators (< 40) compared to colorectal cancers with LINE-1 methylation level ≥40.

Clinical, pathologic or molecular feature	Total N	LINE-1 methylation level		P value^
			
		≥40	< 40	
All cases	869	847	22	
Sex				1.00
Men	394 (45%)	384 (45%)	10 (45%)	
Women	475 (55%)	463 (55%)	12 (55%)	
Age (years)				0.0058
< 60	196 (23%)	186 (22%)	10 (45%)	
60-69	363 (42%)	360 (43%)	3 (14%)	
≥70	310 (36%)	301 (36%)	9 (41%)	
Body mass index (BMI, kg/m^2^)				1.00
< 30	667 (83%)	649 (82%)	18 (86%)	
≥30	141 (17%)	138 (18%)	3 (14%)	
Family history of colorectal cancer				0.80
(-)	657 (76%)	641 (76%)	16 (73%)	
(+)	212 (24%)	206 (24%)	6 (27%)	
Smoking status				0.082
Never	349 (41%)	336 (40%)	13 (59%)	
Past or current	508 (59%)	499 (60%)	9 (41%)	
Tumor location				0.094
Proximal colon (cecum to transverse)	370 (44%)	365 (45%)	5 (25%)	
Distal colon (splenic flexure to sigmoid)	274 (33%)	263 (32%)	11 (55%)	
Rectum	192 (23%)	188 (23%)	4 (20%)	
Disease stage				0.66
I	196 (25%)	193 (25%)	3 (14%)	
II	253 (33%)	246 (32%)	7 (33%)	
III	226 (29%)	218 (29%)	8 (38%)	
IV	103 (13%)	100 (13%)	3 (14%)	
Tumor grade				0.45
Low	777 (91%)	758 (91%)	19 (86%)	
High	79 (9.2%)	76 (9.1%)	3 (14%)	
Mucinous component				0.53
0%	554 (65%)	541 (65%)	13 (59%)	
1-49%	188 (22%)	181 (22%)	7 (32%)	
≥50%	115 (13%)	113 (14%)	2 (9.1%)	
Signet ring cell component				1.00
0%	796 (93%)	775 (93%)	21 (95%)	
1-49%	48 (5.6%)	47 (5.6%)	1 (4.5%)	
≥50%	14 (1.6%)	14 (1.7%)	0	
Crohn's-like reaction				1.00
Absent/mild	584 (90%)	571 (90%)	13 (93%)	
Moderate/severe	62 (9.6%)	61 (9.7%)	1 (7.1%)	
Peritumoral lymphocytic reaction				0.73
Absent/mild	744 (89%)	725 (89%)	19 (86%)	
Moderate/severe	93 (11%)	90 (11%)	3 (14%)	
Tumor infiltrating lymphocytes (TIL)				0.73
Absent/mild	740 (89%)	721 (89%)	19 (86%)	
Moderate/severe	96 (11%)	93 (11%)	3 (14%)	
MSI status				0.76
MSI-low/MSS	728 (85%)	709 (85%)	19 (90%)	
MSI-high	124 (15%)	122 (15%)	2 (9.5%)	
CIMP status				0.14
CIMP-0	408 (47%)	393 (46%)	15 (68%)	
CIMP-low	333 (38%)	327 (39%)	6 (27%)	
CIMP-high	128 (15%)	127 (15%)	1 (4.6%)	
*KRAS *mutation				0.38
(-)	538 (63%)	522 (63%)	16 (73%)	
(+)	319 (37%)	313 (37%)	6 (27%)	
*BRAF *mutation				1.00
(-)	728 (87%)	709 (87%)	19 (90%)	
(+)	108 (13%)	106 (13%)	2 (9.5%)	
*PIK3CA *mutation				0.76
(-)	646 (85%)	627 (84%)	19 (90%)	
(+)	118 (15%)	116 (16%)	2 (9.5%)	
TP53 expression				0.19
(-)	488 (57%)	472 (56%)	16 (73%)	
(+)	371 (43%)	365 (44%)	6 (27%)	
CDKN1A				0.099
Lost	682 (81%)	661 (81%)	21 (95%)	
Expressed	158 (19%)	157 (19%)	1 (4.6%)	
CTNNB1 score*				1.00
0-2 (inactive)	482 (64%)	470 (64%)	12 (63%)	
3-5 (active)	273 (36%)	266 (36%)	7 (37%)	
PTGS2 expression				0.073
(-)	142 (16%)	135 (16%)	7 (32%)	
(+)	719 (84%)	704 (84%)	15 (68%)	
FASN expression				0.50
(-)	742 (88%)	721 (88%)	21 (95%)	
(+)	99 (12%)	98 (12%)	1 (4.6%)	

(%) indicates the proportion of cases with a specific clinical, pathologic or molecular feature among tumors with LINE-1 methylation level ≥ 40 [or tumors with LINE-1 methylation level < 40]. A p value for significance is adjusted to p = 0.0021 by Bonferroni correction for multiple hypothesis testing.

^ p values were based on Fisher's exact test.

* CTNNB1 score was calculated as previously described [24].

CIMP, CpG island methylator phenotype; MSI, microsatellite instability; MSS, microsatellite stable.

## Discussion

In this study, we examined whether clinical, pathologic and molecular variables could potentially explain the wide population variability of LINE-1 methylation in colorectal cancer. Global DNA hypomethylation has been associated with genomic instability, and implicated in the development of various human cancers [3-7,47-52]. To estimate global DNA methylation level, we measured tumor LINE-1 methylation, which has been correlated well with cellular 5-methylcytosine level (i.e., global DNA methylation level) in cancer tissues [10-12]. This is not surprising because LINE-1 retrotransposon constitutes a substantial portion (~17%) of the entire human genome [9]. LINE-1 methylation in colorectal cancer is highly variable [18,19], and is strongly associated with survival among colon cancer patients [20]. Therefore, accurate prediction of tumoral LINE-1 methylation level from clinical and pathological features, if possible, may be clinically useful. Furthermore, recent evidence suggests a non-stochastic component of variable LINE-1 methylation levels in synchronous colorectal cancers (i.e., two or more primary tumors in one individual) [21]. Thus, it is of particular interest to identify clinical, environmental or tumoral factors, if any, which influence LINE-1 methylation in colorectal cancer [53].

Although accumulating evidence has suggested global DNA hypomethylation in human cancers, the mechanisms eliciting this alteration are still unknown. Possible mechanisms include inadequate expression or regulation of DNA methyltransferases, insufficient levels of methyl group donors (i.e., disorder of one-carbon metabolism pathway), aberrant activation of DNA demethylases, and altered expression of chromatin regulators directing DNA methyltransferases [54-56]. The mechanism by which global DNA hypomethylation may confer a poor prognosis (i.e., tumor progression) also remains speculative. Genome-wide DNA hypomethylation has been associated with genomic instability [5], which may confer poor prognosis. Transcriptional dysregulation might be another possible mechanism, and activation of proto-oncogenes, endogenous retroviruses, or transposable elements might affect tumor aggressiveness. A third possible mechanism involves inflammatory mediators and tumor hypoxia; the latter has been associated with genomic DNA hypomethylation [57]. Activation of hypoxia-inducible factor HIF1A has been associated with poor prognosis in colon cancer [58]. A better understanding of relationship between LINE-1 hypomethylation and clinical, pathologic, or molecular feature may shed lights on these biological mechanisms of LINE-1 hypomethylation in human cancer.

The efficiency and precision of bisulfite conversion is very crucial for quantitative assays based on sodium bisulfite treatment of genomic DNA. As the non-CpG cytosine in LINE-1 repetitive sequences has been documented to be rarely methylated [33], we used the non-CpG cytosine as a built-in control for bisulfite conversion efficiency and confirmed successful bisulfite conversion by complete conversion of this cytosine (Figure 1, **arrows**). In addition, we have previously assessed precision of bisulfite treatment and precision of subsequent Pyrosequencing assay to measure LINE-1 methylation in paraffin-embedded colon cancers [32]. We assessed precision of bisulfite conversion by repeating bisulfite treatment. Basically, we performed bisulfite conversion on seven different DNA specimen aliquots from each of 10 different colorectal cancers. Bisulfite-to-bisulfite (between-bisulfite treatment) standard deviation ranged from 0.4-2.4 (median, 1.2), indicating good precision of bisulfite conversion in terms of LINE-1 methylation measurement.

We have discovered the relationship between LINE-1 extreme hypomethylation (< 40) and younger age of onset (< 60 year old). The LINE-1 extreme hypomethylators appear to be separate from the normal distribution of LINE-1 methylation observed in the remaining majority of colorectal cancers. This group of colorectal cancers has not been described previously, because it is necessary to analyze a large number of molecularly and clinically-annotated colorectal cancers with precise LINE-1 methylation data. The relationship of LINE-1 extreme hypomethylation with earlier age of onset and poor prognosis [20] support the presence of a distinct subtype of colorectal cancers with a unique pathogenic mechanism. Nonetheless, additional studies are necessary to confirm our findings. We are currently investigating whether this class of LINE-1 extreme hypomethylators may indeed be driven by different underlying molecular mechanisms than those inducing the normal distribution of LINE-1 methylation seen in the majority of colorectal cancers.

We have also found that the linear regression model explains only 8.4% of variability of LINE-1 methylation, and the 92% of the variability still remained unexplained. In addition, we have shown that multivariate logistic regression models do not significantly predict binary LINE-1 methylation outcomes. Our results imply that LINE-1 methylation in colorectal cancer can vary greatly even after accounting for the clinical, pathologic and molecular features (examined in this study), despite the uniformity within individual patients in cases of synchronous colorectal cancers [21]. Our data could point to several interesting potential hypotheses for underlying biological mechanisms; First, genomic methylation levels and tumor progression could be co-influenced by a variety of environmental factors (e.g., smoking, alcohol, or dietary pattern) for each patient. Second, a stochastic element in the underlying regulatory network could be improperly downregulated according to genomic DNA methylation status. Third, specific genes responsible for tumor behavior (e.g., tumor progression) might be aberrantly regulated in cases of extreme hypomethylation. We await further studies to elucidate the exact mechanisms of LINE-1 hypomethylation in colorectal cancers.

Examination of epigenetic and genetic aberrations is important in cancer research [59-64]. We utilized a quantitative Pyrosequencing assay for LINE-1 methylation, which is robust and can accurately quantify LINE-1 methylation level [10,12]. Pyrosequencing technology can detect subtle differences in average LINE-1 methylation levels among different colon cancer subtypes [e.g., microsatellite instability (MSI)-high vs. microsatellite stable (MSS)] [18,19], emphasizing the importance of the use of an accurate and precise method to measure LINE-1 methylation. Our previous survival data indicates that LINE-1 measurement by Pyrosequencing is highly significantly associated with patient outcome [20]. Considering the high reproducibility of LINE-1 Pyrosequencing [10,12,32], the large variation in LINE-1 methylation levels likely reflects true heterogeneity in global methylation levels among individual tumors. Inter-tumoral biological heterogeneity reflecting the LINE-1 methylation variation is also supported by the highly significant association between LINE-1 methylation level and patient survival [20].

Molecular classification of colorectal cancer based on MSI and the CpG island methylator phenotype (CIMP) status is increasingly important, because MSI and CIMP status reflect global genomic and epigenomic aberrations in tumor cells. Both CIMP-high and MSI-high are inversely associated with LINE-1 hypomethylation, suggesting that CIMP/MSI and genomic hypomethylation may represent different pathways to colorectal cancer. In addition, in non-MSI-high tumors, chromosomal instability (CIN) is correlated with LINE-1 hypomethylation, supporting the possible link between genome-wide hypomethylation and CIN. Further studies are necessary to examine the exact mechanism of how genomic hypomethylation, CIMP, MSI and CIN interact in colorectal cancer development and contribute to colorectal cancer progression.

In the multivariate linear and logistic regression models, we included key molecular events that have been well documented in colorectal cancer [65-68]. Those molecular features include CIMP, MSI and TP53, all of which are related with LINE-1 or global DNA hypomethylation [4-7,18,19,49,50]. In addition, our tumor database has enabled us to include other molecular variables (such as CDKN1A expression, CTNNB1 score, PTGS2 expression, FASN expression, *KRAS *mutation, *BRAF *mutation and *PIK3CA *mutation, all of which have been related with MSI or CIMP), as well as clinical and pathologic variables. Therefore, our multivariate linear and logistic regression analysis was quite comprehensive.

Although the multivariate linear regression model cannot explain a wide variation of tumoral LINE-1 methylation, the model can provide us with useful information on the independent relations of LINE-1 methylation with various clinical, pathologic and molecular features of colorectal cancer. The possible relationship of LINE-1 methylation with signet ring cell component (p = 0.0003) and rectal location (vs. colon; p = 0.0046) are intriguing. The mechanism of this relation remains to be investigated. In contrast, we found no significant difference in LINE-1 methylation between right colon and left colon, which is in agreement with data on LINE-1 methylation in normal colonic mucosa [52,53]. The relation between family history of colorectal cancer and LINE-1 hypomethylation (p = 0.0089) may imply the presence of genetic component in global DNA hypomethylation. Of note, we have previously reported that certain SNPs in one-carbon metabolism genes are not associated with LINE-1 hypomethylation [69]. Nonetheless, any of associations with p > 0.0021 in this current study could be a chance finding due to multiple hypothesis testing. Additional independent studies are necessary to confirm these associations.

## Conclusions

In this study utilizing large database of 869 colorectal cancers, we have shown three main findings; first, LINE-1 extreme hypomethylators skew a distribution of LINE-1 to a non-normal distribution, and constitute a cluster in the residuals plot in multivariate linear regression. Second, LINE-1 extreme hypomethylation is associated with younger age of onset, suggesting the presence of a previously-unrecognized, distinct cancer subtype. Third, LINE-1 methylation level in colorectal cancer varies greatly, even after accounting for various clinicopathologic and other molecular variables. Our results support enormous epigenomic diversity of colorectal cancers in terms of LINE-1 methylation status and a possible subset of mechanistically distinct LINE-1 extreme hypomethylators. These findings may have considerable clinical implications, since LINE-1 hypomethylated colon cancers exhibit aggressive clinical behavior [20].

## Abbreviations

AJCC: American Joint Committee on Cancer; ANOVA: analysis of variance; AUC: area under the curve; CI: confidence interval; CIMP: CpG island methylator phenotype; CIN: chromosomal instability; ICC: intraclass correlation coefficient; LOH: loss of heterozygosity; MSI: microsatellite instability; MSS: microsatellite stable; ROC: receiver operator characteristics; SD: standard deviation; TIL: tumor infiltrating lymphocytes.

## Competing interests

The authors declare that they have no competing interests.

## Authors' contributions

SO conceived the study. YB, CH, KN, NT, KS, ELG, CSF, SO collected data. YB, CH, KN, NT, KS, AH, ESS, DJH, ELG, CSF, SO analyzed and interpreted data. SO drafted the manuscript. All authors read, edited and approved the manuscript.

## Supplementary Material

Additional file 1 **The method summary**. This supplementary table summarizes the methods to evaluate LINE-1 methylation, *KRAS*, *BRAF *and *PIK3CA *mutation, CIMP, MSI, CIN, and expression for TP53, CDKN1A, CTNNB1, PTGS2, and FASN. Click here for file
