# Supplementary material for: Epigenomic diversity of colorectal cancer indicated by LINE-1 methylation in a database of 869 tumors
Source: Mol Cancer. 2010 May 27;9:125. doi: 10.1186/1476-4598-9-125 (PMC2892454; doi:10.1186/1476-4598-9-125)
Supplement: Additional file 1 — The method summary. This supplementary table summarizes the methods to evaluate LINE-1 methylation, KRAS, BRAF and PIK3CA mutation, CIMP, MSI, CIN, and expression for TP53, CDKN1A, CTNNB1, PTGS2, and FASN. [file 1476-4598-9-125-S1.DOC]

# Additional file 1

| Tumor variable | Method | Note | Ref |
| --- | --- | --- | --- |
| LINE-1 methylation | Bisulfite treatment of DNA, and PCR-Pyrosequencing | See detail in text | [19, 32] |
| *KRAS* mutation | PCR-Pyrosequencing | Codons 12 and 13 | [34] |
| *BRAF* mutation | PCR-Pyrosequencing | Codon 600 | [35] |
| *PIK3CA* mutation | PCR-Pyrosequencing | Exons 9 and 20 | [36, 37] |
| CIMP (CpG island methylator phenotype) status | Bisulfite treatment of DNA, and real-time PCR for 8 CIMP-specific promoters (*CACNA1G, CDKN2A*, *CRABP1, IGF2, MLH1, NEUROG1, RUNX3* and *SOCS1*) | CIMP-high was defined as the presence of ≥ 6/8 methylated promoters, CIMP-low as the presence of 1/8-5/8 methylated promoters, and CIMP-0 as the absence (0/8) of methylated promoters. | [30, 31] |
| MSI (microsatellite instability) status | PCR and DNA fragment analysis using 10 microsatellite markers (D2S123, D5S346, D17S250, BAT25, BAT26, BAT40, D18S55, D18S56, D18S67 and D18S487) | MSI-high was defined as the presence of instability in ≥30% of the markers, and MSI-low/microsatellite stability (MSS) as the presence of 0-29% unstable markers. | [38] |
| CIN (chromosomal instability) status | PCR and loss of heterozygosity (LOH) analysis using microsatellite markers (D2S123, D5S346, D17S250, D18S55, D18S56, D18S67 and D18S487) | LOH at each locus was defined as ≥40% reduction of one of two allele peaks in tumor DNA relative to normal DNA. CIN positivity was defined as the presence of LOH in any of the chromosomal segments among 2p, 5q, 17q and 18q. | [39, 40] |
| TP53 expression | Immunohistochemistry | TP53+ was defined as the presence of moderate or strong staining in ≥50% of tumor cells. | [42] |
| CDKN1A (p21)  expression | Immunohistochemistry | CDKN1A loss was defined as no or weak staining in tumor cell nuclei or the presence of moderate staining in <20% of tumor cells. | [43, 44] |
| CTNNB1 (β-catenin) score | Immunohistochemistry | CTNNB1 score was calculated as the sum of nuclear score (0, 1+, 2+), cytoplasmic score (0, 1+, 2+), and membrane score (0 if intact; +1 if lost). | [24] |
| PTGS2 (COX-2) expression | Immunohistochemistry | PTGS2+ was defined as the presence of weak to strong expression. | [22, 38] |
| FASN expression | Immunohistochemistry | FASN+ was defined as the presence of strong expression. | [38, 45] |
